# Greater socioenvironmental risk factors and higher chronic pain stage are associated with thinner bilateral temporal lobes

**DOI:** 10.1002/brb3.3330

**Published:** 2023-11-20

**Authors:** Lisa H. Antoine, Jared J. Tanner, Angela M. Mickle, Cesar E. Gonzalez, Daniel A. Kusko, Kristen Allen Watts, Deanna D. Rumble, Taylor L. Buchanan, Andrew M. Sims, Roland Staud, Song Lai, Hrishikesh Deshpande, Brandis Phillips, Thomas W. Buford, Edwin N. Aroke, David T. Redden, Roger B. Fillingim, Burel R. Goodin, Kimberly T. Sibille

**Affiliations:** ^1^ Department of Psychology University of Alabama at Birmingham Birmingham Alabama USA; ^2^ Department of Clinical and Health Psychology University of Florida Gainesville Florida USA; ^3^ Pain Research & Intervention Center of Excellence University of Florida Gainesville Florida USA; ^4^ Department of Physical Medicine & Rehabilitation University of Florida Gainesville Florida USA; ^5^ Heersink School of Medicine University of Alabama at Birmingham Birmingham Alabama USA; ^6^ Department of Psychology and Counseling University of Central Arkansas Conway Arkansas USA; ^7^ Center for Exercise Medicine University of Alabama at Birmingham Birmingham Alabama USA; ^8^ Department of Biostatistics University of Alabama at Birmingham Birmingham Alabama USA; ^9^ Department of Medicine University of Florida Gainesville Florida USA; ^10^ Department of Radiation Oncology University of Florida Gainesville Florida USA; ^11^ Department of Radiology University of Alabama at Birmingham Birmingham Alabama USA; ^12^ Department of Accounting & Finance North Carolina A&T State University Greensboro North Carolina USA; ^13^ Department of Medicine − Division of Gerontology, Geriatrics, and Palliative Care University of Alabama at Birmingham Birmingham Alabama USA; ^14^ Birmingham/Atlanta Geriatric Research, Education, and Clinical Center Birmingham VA Medical Center Birmingham Alabama USA; ^15^ School of Nursing University of Alabama at Birmingham Birmingham Alabama USA; ^16^ Department of Biostatistics University of Alabama at Birmingham Birmingham Alabama USA; ^17^ Department of Community of Dentistry and Behavioral Sciences University of Florida Gainesville Florida USA; ^18^ Department of Anesthesiology Washington University, Washington University Pain Center St. Louis Missouri USA

**Keywords:** Alzheimer's disease risk, brain imaging, chronic pain, health disparities, osteoarthritis

## Abstract

Introduction: Previous research indicates ethnic/race group differences in pain and neurodegenerative diseases. Accounting for socioenvironmental factors reduces ethnic/race group differences in clinical and experimental pain. In the current study sample, we previously reported that in individuals with knee pain, ethnic/race group differences were observed in bilateral temporal lobe thickness, areas of the brain associated with risk for Alzheimer's disease, and related dementias. The purpose of the study was to determine if socioenvironmental factors reduce or account for previously observed ethnic/race group differences and explore if a combined effect of socioenvironmental risk and chronic pain severity on temporal lobe cortices is evident.

Methods: Consistent with the prior study, the sample was comprised of 147 adults (95 women, 52 men), 45–85 years of age, who self‐identified as non‐Hispanic Black (*n* = 72) and non‐Hispanic White (*n* = 75), with knee pain with/at risk for osteoarthritis. Measures included demographics, health history, pain questionnaires, cognitive screening, body mass index, individual‐ and community‐level socioenvironmental factors (education, income, household size, marital and insurance status, and area deprivation index), and brain imaging. We computed a summative socioenvironmental risk index. Results: Regression analyses showed that with the inclusion of socioenvironmental factors, the model was significant (*p* < .001), and sociodemographic (ethnic/race) group differences were not significant (*p =* .118). Additionally, findings revealed an additive stress load pattern indicating thinner temporal lobe cortices with greater socioenvironmental risk and chronic pain severity (*p =* .048).

Implications: Although individual socioenvironmental factors were not independent predictors, when collectively combined in models, ethnic/race group differences in bilateral temporal lobe structures were not replicated. Further, combined socioenvironmental risk factors and higher chronic pain severity were associated with thinner bilateral temporal lobes.

## Graphical abstract

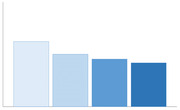

## 1 INTRODUCTION

Ethnic/race group disparities have been reported across experimental and clinical pain (Kim et al., 2017; Vaughn et al., 2019, 2018). However, building research shows that when socioenvironmental factors are accounted for, ethnic/race group differences wane (Mickle et al., 2023; Mullins et al., 2022; Zajacova et al., 2022). In fact, our recent findings, based on data from two different studies, including participants for the current study sample, show that with the inclusion of socioenvironmental factors in analyses, previously reported ethnic/race group differences in experimental and clinical pain are reduced (Mickle et al., 2023).

Ethnic/race group differences in Alzheimer's disease and related dementias (ADRD) have also been reported. Non‐Hispanic Black (NHB) adults experience an incidence of ADRD up to 63% higher than non‐Hispanic White (NHW) adults (Mayeda et al., 2016; Mehta & Yeo, 2017; Steenland et al., 2016). Prior research shows possible links between chronic pain, temporal lobe brain structures, and dementia risk (Ezzati et al., 2019; Whitlock et al., 2017; Yamada et al., 2019; Zhao et al., 2023). We previously investigated in the current study sample the relationships between chronic pain, cognition, temporal lobes (an area of the brain associated with ADRD risk), and possible ethnic/race group differences (Tanner et al., 2021). We observed that in individuals with high chronic pain stage, NHB adults had significantly thinner temporal lobe gray matter than NHW adults. Importantly, the NHB adults also had greater sociodemographic risk factors compared to their NHW peers (Tanner et al., 2021). Consistent with the allostatic load conceptualization, we hypothesized that greater socioenvironmental stress adds an additional physiological and neurobiological “load” to the biological burden of chronic pain contributing to higher allostatic load which was indicated by thinner temporal lobe brain structures (McEwen, 2015; McEwen, 1998; Mickle et al., 2023). Following our publication and aligning with our interpretation, a population‐based study indicated that greater socioenvironmental risk based on an additive social determinants of health (SDOH) index was associated with greater osteoarthritis disease severity (Rethorn et al., 2022).

Informed by the National Institute on Aging and the National Institute on Minority Health and Health Disparities) Health Disparities Research Frameworks (Alvidrez et al., 2019; Hill et al., 2015), we were interested in determining if individual and community‐level socioenvironmental factors would explain the previously observed differences we reported in temporal lobe brain structures in the same study sample. Individual‐level socioenvironmental factors include factors such as education, income, marital status, insurance status, and number of people living in the household. Adults with greater socioenvironmental risk, that is, an income ≤$25,000, less than a high school education, no insurance or domestic partner, and/or unemployed experience more disabling pain compared to those with lower socioenvironmental risk (Allen‐Watts et al., 2021; Janevic et al., 2017; Mullins et al., 2022; Portenoy et al., 2004).

Community‐level socioenvironmental factors include measures such as the Area Deprivation Index (ADI) and the Centers for Disease Control Social Vulnerability Index (SVI). Both measures are derived from housing zip codes. The ADI reflects community‐level resources and experiences, while the SVI captures infrastructure and vulnerability particularly for disaster management (Flanagan et al., 2011; Kind & Buckingham, 2018; Singh, 2003). The ADI is associated with poor quality sleep, higher inflammation, and worse chronic pain symptoms in individuals with chronic low back pain (Dembowski et al., 2022; Rumble et al., 2021).

We investigate whether a comprehensive array of socioenvironmental factors in combination with chronic pain severity accounts for the bilateral temporal lobe cortical thickness differences observed between NHB and NHW adults with knee pain with or at risk for osteoarthritis. First, we evaluate the relationships between individual and community‐level socioenvironmental factors and bilateral temporal lobe cortical thickness. Second, we test whether previously observed ethnic/race group differences in bilateral temporal lobe cortical thickness are retained after including individual and community‐level socioenvironmental factors in the analyses. Third, we replicate a version of the SDOH risk index from a population‐based study and determine if the combined contributions of socioenvironmental risk and chronic pain severity contribute additive stress, i.e., greater allostatic load, as indicated by thinner temporal lobes.

## 2 MATERIALS AND METHODS

### 2.1 Design

Understanding Pain and Limitations in Osteoarthritic Disease‐2 (UPLOAD‐2) was a prospective study conducted at the University of Florida (UF) and the University of Alabama at Birmingham (UAB). The baseline data in the current study were collected from August 2015 through May 2017.

### 2.2 Participants

The UPLOAD‐2 study examined biopsychosocial factors in NHB and NHW adults between 45 and 85 years of age with and without knee pain. Inclusion for the current study was limited to those individuals with knee pain and who had imaging data to address research questions. Exclusion criteria included rheumatologic conditions, knee replacement surgery, neurological diseases, cardiovascular or peripheral arterial disease, psychiatric disorders requiring hospitalization within the past year, pregnant or nursing, and/or unable to complete magnetic resonance imaging (MRI). The inclusion and exclusion criteria were also described for the current study sample in our prior publication (Tanner et al., 2021). The Institutional Review Boards at UF and UAB reviewed and approved the study protocol. All participants provided informed verbal and written consent according to the Declaration of Helsinki and received compensation for their participation. This study follows the Strobe Checklist guidelines (von Elm E et al., 2008). Measures specific to the current analysis are described.

### 2.3 Measures

#### 2.3.1 Descriptive

Data collected include age, ethnicity/race, and sex. Height and weight measures were taken, and body mass index (BMI) was calculated.

### 2.4 Socioenvironmental

Individual‐level data included education level, income, socioeconomic status (SES), number of people living in the household (household number), employment status, insurance status, and marital status.

Education levels ranged from 1 to 6, where 1 indicates did not complete high school, 2 indicates obtained high school diploma, 3 indicates 2‐year college degree, 4 indicates 4‐year college degree, 5 indicates master's degree, and 6 indicates doctoral degree or equivalent.

Income levels ranged from 1 to 10, where 1 is $0–$9,999, 2 is $10,000–$19,999, 3 is $20,000–$29,999, 4 is $30,000–$39,999, 5 is $40,000–$49,999, 6 is $50,000–$59,999, 7 is $60,000–$79,999, 8 is $80,000–$99,999, 9 is $100,000–$149,999, and 10 is $150,000 or higher.

SES was computed by averaging the *z* score of the education level (categorical range: 1 [did not complete high school] to 6 [doctoral degree]) and *z* score of income level (categorical range 1 [$0–$9999] to 10 [$150,000 or higher]) into a *z* score composite value. SES composite score was used in all analyses.

Employment status included 0 = not working, temporarily laid off, student, disabled, other, and 1 = working, retired.

Insurance status was either 0 = no insurance or 1 = some type of insurance.

Marital status included 0 = widowed, divorced, separated, and never married and 1 = married or living with partner.

Community‐level data were determined by the ADI. The ADI is derived from the Neighborhood Atlas (Kind & Buckingham, 2018). The participant's address was used to assign a census block group (nine‐digit zip code). National ADI scores span 1–100 with higher scores representing higher deprivation.

The socioenvironmental risk index was developed based on an index that was previously described (Rethorn et al., 2022). Poverty level was determined from the U.S. federal poverty guidelines based on income and household number. Imputation was used for eight participants missing one of the measures replacing with data from another time point. Each variable was assigned a 0 for protective or 1 for risk based on evidence‐based ranges and then summed with a score range of 0–6 (Table 1). Median value determined low risk = 0–2 and high risk = 3–6.

**TABLE 1:** Socioenvironmental risk index.

Socioenvironmental factor	Score
Education	
Greater than high school	0
High school or less	1
Poverty level^a^	
No	0
Yes	1
Marital status^b^	
Married or living with partner	0
Widowed, divorced, separated, and never married	1
Employment status	
Employed or retired	0
Laid off/on leave, looking for work, disabled, student, and other	1
Insurance status	
Insured	0
Not insured	1
Area deprivation index (ADI)	
Lower 80%	0
Upper 20%	1

*Note*: Index modeled from prior publication (Rethorn et al., 2022).

^a^ For missing income or household number, poverty was imputed based on income and household number reported at the relevant time point.

^b^ For missing marital status, imputation was based on marital status reported at relevant time point or based on the number of people living in the household when individuals only reported one (self).

### 2.5 Cognitive

The Montreal Cognitive Assessment (MoCA) is a brief screening measure that assesses cognitive functioning across seven domains: visuospatial/executive, naming, attention, language, abstraction, delayed recall, and orientation (Rossetti et al., 2011). The total score range is 0–30. We and others have reported that the MoCA is associated with chronic pain severity and functional limitations, including a subset of participants from the UPLOAD2 study (Cardoso et al., 2021; Crowley et al., 2022; Ferreira Kdos et al., 2016).

### 2.6 Chronic pain severity

Chronic pain stage was computed specific to *frequency* (number of days per week knee pain is experienced), *intensity* (based on the Graded Chronic Pain Scale [GCPS] characteristic pain intensity), *duration of knee pain* (length of time experiencing knee pain), and *total number of body sites* (Tanner et al., 2021). The GCPS assesses knee pain severity in the past 6 months (Von Korff et al., 1992). Characteristic pain intensity was determined by taking the mean of current, average, and worst pain using a scale of 0 (no pain) to 10 (worse pain ever) and multiplying the result by 10. Total pain sites were determined by a question assessing pain experienced on more days than not over a 3‐month or greater period. There were a total of 28 body sites (14 on each side) with scores ranging from 0 to 28.

Chronic pain stage is calculated by scoring each domain (frequency, intensity, duration, and total number of pain sites) as 0 if below the median score for the measure or 1 if above the median score. The four domains are totaled, and a score range of 1–5 is determined with higher scores indicating higher chronic pain severity (Sibille et al., 2017, 2016). For group comparisons, consistent with our prior study, the low chronic pain stage is identified as stages 1 and 2, and the high chronic pain stage is defined stages 4 and 5 (Tanner et al., 2021).

### 2.7 Brain imaging

MRI data were collected using a 3 Tesla Philips Achieva (eight‐channel head coil at UAB and 32‐channel at UF) to obtain three‐dimensional magnetization‐prepared rapid acquisition gradient‐echo (MP‐RAGE) images. The parameters for the MP‐RAGE images follow echo time = 3.2 ms, repetition time = 7.0 ms, flip angle = 8 degrees, 1 mm iso voxels, and field of view = 240 × 240 × 176, sagittal acquisition. MP‐RAGE images were processed with FreeSurfer 6.0 (Fischl et al., 2004; FreeSurfer, 2012; Klein & Tourville, 2012; Salat et al., 2004). FreeSurfer has been shown to be reliable among various scanner manufacturers and field strengths (Han et al., 2006; Reuter et al., 2012). Mean native space cortical thickness values in millimeters across the left and right temporal lobes specific to the entorhinal cortex, fusiform gyrus, and inferior and middle temporal gyri were averaged (Parikh et al., 2011). These temporal lobe regions include areas that have been previously linked to chronic pain (Magon et al., 2018; Schwedt et al., 2015; Tanner et al., 2021), AD pathology and related cognitive decline (Jack et al., 2015), and progression to mild cognitive impairment (Petersen et al., 2019).

### 2.8 Statistical analysis

Data were checked for absence, normality, and outliers using visual inspection of univariate plots frequency tables, and using the Shapiro–Wilk test for normality. Bilateral temporal lobes were normally distributed (Shapiro–Wilk *p* value = .827). Although other variables failed to fit the assumption of normality (Shapiro–Wilk *p* values < .001), given the sample size and visual inspection, we used parametric statistical approaches where described.

Variable selection for the inclusion of covariates was completed by correlational analyses between age, sociodemographic group, study site, sex, BMI, and MoCA total score with temporal lobes cortical thickness. As there were no associations between sex and temporal lobe brain structures (*ρ* = 0.02, *p =* .800), sex was not included as a covariate in the study models. The following variables were included as covariates in the study models: age, sociodemographic groups, study site, BMI, and MoCA. Age, BMI, and MoCA total were continuous variables. Ethnic/race group and study site were categorical: 1 (NHB) and 2 (NHW); 1 (UF) and 2 (UAB). The following analyses were completed:

1. Determine relationships between socioenvironmental factors and temporal lobe cortical thickness. A Spearman correlation was computed between individual and community‐level socioenvironmental variables and bilateral temporal lobe thickness. To optimize model design, we used SES which is a combined measure of education and income. Regression analysis was completed with the socioenvironmental factors and covariates.
2. Determine whether previously observed ethnic/race group differences in temporal lobes cortical thickness are retained after adding additional socioenvironmental factors to the model. The prior study analysis was replicated with additional socioenvironmental factors included (Tanner et al., 2021).

A regression analysis was completed with the socioenvironmental factors, covariates, and chronic pain stage.

3. Determine whether low and high socioenvironmental risk and low and high chronic pain stage account for the ethnic/race group differences observed in our prior publication (Tanner et al., 2021). Low and high socioenvironmental risk replaced the ethnic/race group variable as reported in our prior publication. A two‐way analysis of covariance (ANCOVA) was completed. Thus, low and high socioenvironmental risk and low chronic pain stage (stages 1 and 2) and high chronic pain stage (stages 4 and 5) were investigated with matching covariates from the prior study which included age, BMI, study site, SES, and MoCA total score (Tanner et al., 2021). Due to small group sample sizes, a post hoc linear regression was also completed with a combined low/low, low/high, high/low, and high/high socioenvironmental risk and chronic pain stage variables, including the covariates age, study site, and BMI.

All statistical tests were two‐tailed, and the alpha level used to determine significance was *p* < .05. Statistical analyses were completed with IBM SPSS v 28.0 and SAS v 9.4 (Cary, NC) (Corp, 2017; STAT‐SAS, 2010) and run independently by two co‐authors for internal rigor and confirm reproducibility.

## 3 RESULTS

### 3.1 Participant characteristics

A total of 147 individuals were included with a mean (SD) age of 58.3 (±8.0 years), 63% of the sample were from UF, 65% were female, and 56% had post‐high school education (Table 2). More than 40% of the sample were married or living with a partner. Most participants (∼86%) had some type of health insurance. The mean (SD) ADI was 65.4 (±23.1) indicating a sample with higher community‐level deprivation. Study sites did not differ by sociodemographic variables.

**TABLE 2:** Baseline characteristics.

		Sociodemographic groups		
	Total sample (*n* = 147)	NHB (*n* = 72)	NHW (*n* = 75)	*p*
Age, mean ± SD	58.3 ± 8.0	56.3 ± 6.3	60.3 ± 8.9	**.009**
Study site, *n* (%)				.384
UF	93 (63.3)	43 (59.7)	50 (66.7)	
UAB	54 (36.7)	29 (40.3)	25 (33.3)	
Sex, *n* (%)				.225
Male	52 (35.4)	29 (40.3)	23 (30.7)	
Female	95 (64.6)	43 (59.7)	52 (69.3)	
BMI, mean ± SD	31.3 ± 6.4	31.9 ± 5.9	30.7 ± 6.8	.174
MoCA, mean ± SD	24.2 ± 3.5	22.7 ± 3.7	25.8 ± 2.4	**<.001**
Socioenvironmental factors				
Education, *n* (%)				**.002**
Some school	10 (6.8)	8 (11.1)	2 (2.6)	
High school degree	55 (37.4)	32 (44.4)	23 (30.7)	
Two‐year college degree	25 (17.0)	12 (16.7)	13 (17.3)	
Four‐year college degree	32 (21.8)	11 (15.3)	21 (28.0)	
Master's degree	18 (12.2)	7 (9.7)	11 (14.7)	
Doctoral degree	7 (4.8)	2 (2.8)	5 (6.7)	
Income, *n* (%)				**<.001**
$0–$9,999	34 (23.1)	24 (33.3)	10 (13.3)	
$10,000–$19,999	17 (11.6)	11 (15.3)	6 (8.0)	
$20,000–$29,999	21 (14.3)	12 (16.6)	9 (12.0)	
$30,000–$39,999	6 (4.1)	4 (5.5)	2 (2.7)	
$40,000–$49,999	12 (8.2)	3 (4.2)	9 (12.0)	
$50,000–$59,999	16 (10.9)	5 (6.9)	11 (14.7)	
$60,000–$79,999	13 (8.8)	4 (5.5)	9 (12.0)	
$80,000–$99,999	9 (6.1)	4 (5.5)	5 (6.7)	
$100,000–$149,999	11 (7.5)	2 (2.8)	9 (12.0)	
$150,000 or higher	5 (3.4)	1 (1.4)	4 (5.3)	
Not reported	3 (2.0)	2 (2.8)	1 (1.3)	
Household number, median [IQR]	2.0 [2.0]	2.0 [2.0]	2.0 [2.0]	.963
Employment status, *n* (%)				.052
Working/retired	101 (68.7)	44 (61.1)	57 (76.0)	
Not working/not retired	46 (31.3)	28 (38.9)	18 (24.0)	
Current insurance, *n* (%)	127 (86.4)	61 (84.7)	75 (100.0)	.562
Marital status, *n* (%)				**<.001**
Married/partner	65 (44.2)	21 (29.2)	44 (58.7)	
Divorced/widowed/single	80 (54.4)	49 (68.0)	31 (41.3)	
Not reported	2 (1.4)	2 (2.8)	0 (0.0)	
ADI national, mean ± SD	65.4 ± 23.1	73.9 ± 20.3	57.3 ± 22.7	**<.001**
Chronic pain severity				
Total pain sites, median [IQR]	5.0 [5.0]	5.0 [5.0]	5.0 [4.0]	.475
GCPS pain intensity, mean ± SD	54.1 ± 23.3	66.1 ± 21.3	42.7 ± 19.1	**<.001**
Chronic pain stage median [IQR]	2.0 [2.0]	2.0 [2.0]	2.0 [1.0]	**.010**

Abbreviations: ADI, Area Deprivation Index; BMI, body mass index; GCPS, Graded Chronic Pain Scale; IQR, interquartile range; MoCA, Montreal Cognitive Assessment; NHB, non‐Hispanic Black; NHW, non‐Hispanic White; SD, standard deviation; UAB, University of Alabama at Birmingham; UF, University of Florida.

As this study was limited to individuals with knee pain who completed MRIs in the UPLOAD‐2 dataset, we ran comparisons between participants reporting knee pain who completed MRI (*n* = 147) and those who did not complete MRI (*n* = 41). Individuals who did not complete an MRI reported a higher ADI (*p =* .037) and lower SES (*p* < .001) compared to those individuals completing MRIs. No other sociodemographic differences were observed, for example, age, sex, ethnicity/race, chronic pain stage, and BMI.

NHB participants were younger, reported a lower level of education and income, were single, lived in more disadvantaged areas, and scored lower on the MoCA (Table 2). The imbalance in sociodemographic factors results in an incomplete representation of each ethnic/race group. Thus, the term sociodemographic group was used.

### 3.2 Socioenvironmental factors and bilateral temporal lobe thickness

Model 1 in Table 3 provides Spearman's correlational findings between SES (*p =* .255), employment (*p =* .784), insurance status (*p =* .946), marital status (*p =* .753), and ADI national (*p =* .095) and bilateral temporal lobes cortical thickness. In an adjusted analysis, the overall regression model was significant (*F* (11,131) = 4.17, *p* < .001) with younger age, the UAB study site, and lower BMI associated with thicker temporal lobe cortical structures (Table 3 [Aim1: Model 1]).

**TABLE 3:** Multiple regressions predicting bilateral temporal lobe thickness from socioenvironmental factors and chronic pain stage.

Predictor	*b*	95% CI	*β*	95% CI	Unique *R* ^2^	95% CI	rho	Fit	ΔFit
Aim 1: Model 1 (intercept)									
	3.18**	[2.89, 3.46]							
Age	−0.01**	[−0.01, −0.00]	−0.38	[−0.56, −0.20]	0.10**	[0.01, 0.19]	−0.17*		
Sociodemographic group	0.04	[−0.01, 0.09]	0.16	[−0.02, 0.35]	0.02	[−0.02, 0.05]	0.17*		
Study site	0.07**	[0.03, 0.11]	0.26	[0.11, 0.42]	0.06**	[−0.01, 0.13]	0.22**		
BMI	−0.01**	[−0.01, −0.00]	−0.35	[−0.51, −0.19]	0.11**	[0.02, 0.19]	−0.23**		
MoCA total	0.00	[−0.00, 0.01]	0.05	[−0.13, 0.24]	0.00	[−0.01, 0.01]	0.10		
SES	0.01	[−0.02, 0.04]	0.09	[−0.12, 0.31]	0.00	[−0.01, 0.02]	0.10		
Household number	−0.02	[−0.03, 0.00]	−0.15	[−0.34, 0.04]	0.01	[−0.02, 0.05]	0.01		
Employment status	−0.01	[−0.06, 0.04]	−0.02	[−0.21, 0.16]	0.00	[−0.01, 0.01]	−0.02		
Insurance status	0.02	[−0.05, 0.08]	0.04	[−0.13, 0.21]	0.00	[−0.01, 0.01]	−0.01		
Marital status	0.01	[−0.04, 0.06]	0.04	[−0.16, 0.24]	0.00	[−0.01, 0.01]	0.03		
ADI national	−0.00	[−0.00, 0.00]	−0.03	[−0.21, 0.14]	0.00	[−0.01, 0.01]	−0.14		
								*R* ^2^ = 0.259**	
								95% CI [0.08,0.32]	
Aim 2: Model 2 (intercept)	3.20**	[2.91, 3.49]							
Age	−0.01**	[−0.01, −0.00]	−0.39	[−0.57, −0.21]	0.10**	[0.02, 0.19]	−0.17*		
Sociodemographic group	0.04	[−0.01, 0.08]	0.15	[−0.04, 0.34]	0.01	[−0.02, 0.05]	0.17*		
Study site	0.07**	[0.03, 0.11]	0.25	[0.10, 0.41]	0.06**	[−0.01, 0.13]	0.22**		
BMI	−0.01**	[−0.01, −0.00]	−0.34	[−0.50, −0.18]	0.10**	[0.01, 0.18]	−0.23**		
MoCA total	0.00	[−0.00, 0.01]	0.06	[−0.13, 0.24]	0.00	[−0.01, 0.01]	0.10		
SES	0.01	[−0.02, 0.04]	0.07	[−0.15, 0.29]	0.00	[−0.01, 0.02]	0.10		
Household number	−0.02	[−0.04, 0.00]	−0.15	[−0.34, 0.04]	0.01	[−0.02, 0.05]	0.01		
Employment status	−0.01	[−0.06, 0.04]	−0.02	[−0.21, 0.16]	0.00	[−0.00, 0.01]	−0.02		
Insurance status	0.02	[−0.04, 0.08]	0.05	[−0.12, 0.22]	0.00	[−0.01, 0.01]	−0.01		
Marital status	0.01	[−0.04, 0.06]	0.04	[−0.16, 0.24]	0.00	[−0.01, 0.01]	0.03		
ADI national	0.00	[−0.00, 0.00]	−0.04	[−0.22, 0.13]	0.00	[−0.01, 0.01]	−0.14		
Chronic pain stage	−0.01	[−0.03, 0.01]	−0.08	[−0.24, 0.09]	0.00	[−0.01, 0.02]	−0.15		
								*R* ^2^ = 0.264**	Δ*R* ^2^ = 0.005
								95% CI [0.08, 0.32]	95% CI [−0.01, 0.02]

*Note*: *N* = 143. *b* = unstandardized regression weight. *β* = standardized regression weight. Unique *R* ^2^ = semi‐partial correlation squared. rho = zero‐order Spearman's correlation.

Abbreviations: ADI National, National Percentile Area Deprivation Index; BMI, body mass index; CI, confidence interval; MoCA total, Montreal Cognitive Assessment total score unadjusted for education; SES, socioeconomic status (education/income). *p < .05; **p < .01.

### 3.3 Chronic pain stage, socioenvironmental factors, and bilateral temporal lobes thickness

Significant associations between chronic pain stage, socioenvironmental factors, and bilateral temporal lobes cortical thickness were indicated (*F* (12,130) = 3.89, *p* < .001). Of note, with the inclusion of additional socioenvironmental factors, sociodemographic (ethnic/race) groups were not a significant predictor (*p =* .118) as previously observed (Tanner et al., 2021). Younger age, the UAB study site, and lower BMI were related to thicker temporal lobe cortical structures (Table 3 [Aim 2: Model 2]).

### 3.4 Low/high socioenvironmental risk and low/high chronic pain stage

The overall model for the ANCOVA analysis was significant; however, the group effect was not significant (*p =* .245, partial *ƞ* ^2^ = 0.043). Group sample sizes were a limitation: low socioenvironmental risk and low chronic pain stage, *n* = 35; low risk and high pain stage, *n* = 17; high risk and low pain stage, *n* = 24; and high risk and high pain stage, *n* = 24.

**FIGURE 1:**
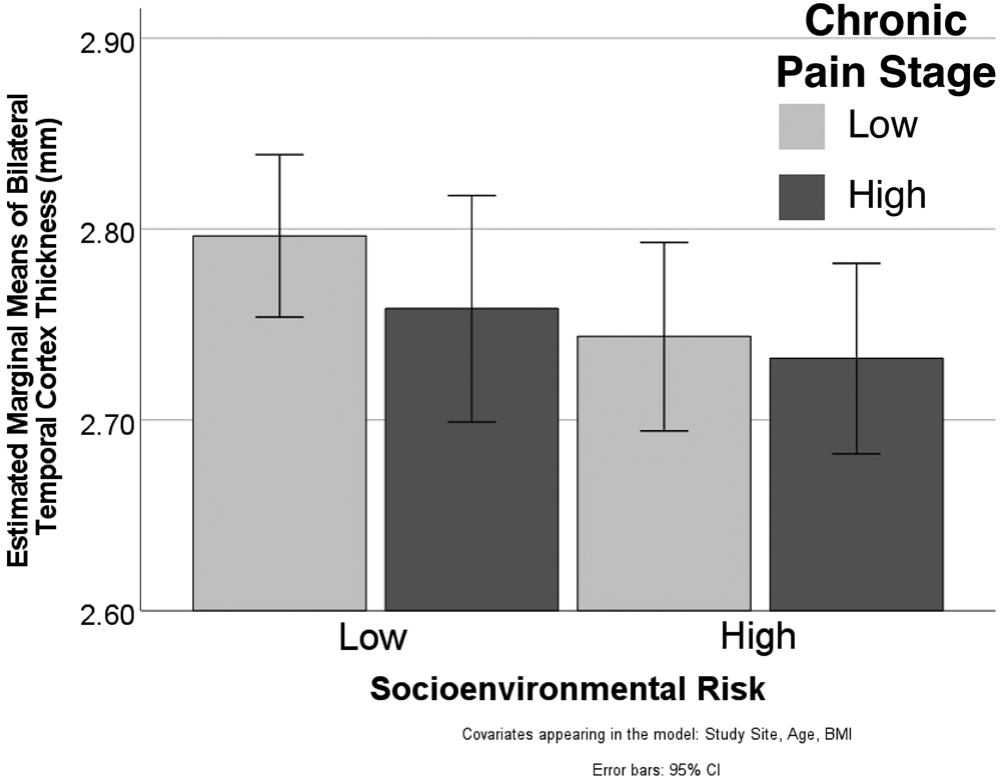
Bilateral temporal cortex thickness for socioenvironmental risk and chronic pain stage groups. Covariates appearing in the model: study site, age, and body mass Index (BMI). Overall model (*F* 3.82, *p* = .006) group (*p* = .048). Error bars: 95% confidence interval [CI]. *n* = 100.

A post hoc linear analysis with the four groups was completed. The overall model was significant (*F* (4,95) = 3.82, *p =* .006) as well as the group (*p =* .048). The pattern of results indicates an inverse additive relationship with thicker temporal lobe cortices in individuals with lower socioenvironmental risk and lower chronic pain stage and thinner temporal lobe cortex in individuals with higher socioenvironmental risk and higher chronic pain stage (Figure 1).

## 4 DISCUSSION

The purpose of the study was to extend previous findings by examining if a comprehensive array of individual and community‐level socioenvironmental factors accounted for the differences observed in temporal lobes cortical thickness between NHB and NHW adults with knee pain (Tanner et al., 2021). Although socioenvironmental factors were not independent predictors, when collectively combined in statistical models, previously observed ethnic/race group differences in temporal lobe structures were no longer indicated. Thinner bilateral temporal lobes were associated with older age, study site location, and higher BMI. Importantly, we show an additive inverse relationship between socioenvironmental risk factors, chronic pain severity, and temporal lobe structures. These findings replicate and extend previously reported additive, dose‐response pattern between an SDOH index and greater osteoarthritis severity in a population‐based study (Rethorn et al., 2022). Findings convey the importance of considering socioenvironmental factors when investigating health disparities in chronic pain and ADRD risk.

Previous research indicates that NHB Americans have heightened risks of ADRDs relative to NHW Americans (Mayeda et al., 2016; Mehta & Yeo, 2017; Steenland et al., 2016; Yaffe et al., 2013). In the current study, with the inclusion of an array of socioenvironmental factors in the model, sociodemographic (ethnic/race) groups are not a significant predictor. Age, BMI, and socioenvironmental factors such as education and income have been consistently associated with brain structure (Davidson & McEwen, 2012; Elbejjani et al., 2017; Peters, 2006; Raji et al., 2010; Tanaka et al., 2020). The study site was also a significant predictor. In addition to possible differences attributed to study site scanners, the differing testing and living environments specific to the study sites could also be a contributing factor. Other variables such as discrimination have been associated with disparities in brain structure and warrant consideration in future investigations (Fani et al., 2022).

There is ample evidence that chronic pain is linked to altered gray matter throughout the brain (Alshuft et al., 2016; Bushnell et al., 2013; Davis & Moayedi, 2013). Similarly, socioenvironmental factors influence brain structure (Elbejjani et al., 2017; Fani et al., 2022). We previously demonstrated differing bilateral temporal lobe brain structures in individuals with similar levels of chronic pain severity (Tanner et al., 2021). The pattern observed in NHB adults who also reported greater socioenvironmental risk was consistent with the hormesis, nonlinear, inverted‐U pattern (Agathokleous & Calabrese, 2019; Calabrese, 2014; Li et al., 2019; Mickle & Sibille, 2023). Comparatively, the NHW adults with a similar level of chronic pain severity and lower socioenvironmental risk had a statistically and clinically thicker bilateral temporal lobe brain structure, suggesting their temporal lobes were still in an “adaptive stage” to the chronic pain stimuli (Tanner et al., 2021).

In the current study, we found that although individual socioenvironmental factors were not significant predictors in the study models, the previously observed sociodemographic (ethnic/race) group differences in the temporal lobe bilateral brain structures were no longer significant with the combination of socioenvironmental factors included. Comparing the variance for the two studies, in the first study, in Model 3 in Table 3, sociodemographic (ethnic/race) group was significant at *p =* .012 and the *R* ^2^ value = 0.248 (Tanner et al., 2021). In the current analysis, sociodemographic group is not significant *p =* .118 and the R^2^ value = 0.264. These findings are highly relevant for helping move health disparities research forward as they indicate the importance of shifting from focusing solely on ethnic/race group differences to identifying the socioenvironmental factors contributing to poor health outcomes.

A novel finding, bilateral temporal lobe differences were indicated in an additive fashion based on a combination of greater socioenvironmental risk and greater chronic pain stage. Results align with the hypothesized interpretation consistent with the allostatic load model, and physiological and neurobiological systems are adaptive to stress until the cumulative load exceeds functional capacity (McEwen, 1998, 2015). Hence, individuals with “combined loads” of high socioenvironmental risk and high chronic pain stage show greater neurobiological “load” as indicated by a pattern of thinner temporal lobe brain structures than their peers with lower levels of socioenvironmental risk and lower levels of chronic pain stage (Mickle et al., 2023).

Strengths of the study include sample size of participants with brain imaging data (Szucs & Ioannidis, 2020); the incorporation of two levels of analysis, socioenvironmental and biological, and two levels of influence, individual and community (Alvidrez et al., 2019; Hill et al., 2015; Patel et al., 2022); and a balanced representation of NHB and NHW adults. Limitations also warrant acknowledgment. First, as ethnic/race groups differed on a number of socioenvironmental variables, increasing diversity within groups and matching participants on sociodemographic factors is needed. Second, there were socioeconomic differences between participants who did and did not complete MRI. The effects of this bias are unknown. Future prospective studies should be designed to increase participation of those who have more limited social and economic resources. Third, participants may have had unreported or undiagnosed conditions associated with cortical thinning (Chen et al., 2020; Galovic et al., 2019; Pletcher et al., 2023). Fourth, the number of channels for the brain imaging coils was less for UAB (eight‐channel) compared to UF (32‐channel) which has some reported differences although differences in channels for the brain imaging coils are not uncommon in multisite studies (Parikh et al., 2011). While the channel‐related factors may contribute to some of the site differences observed, other factors also contribute such as testing site differences and environmental factors as site differences are also common with non‐MRI findings in the UPLOAD‐2 study (Bartley et al., 2019; Booker et al., 2020). Fifth, we conceptualized work status to include those individuals reporting being retired which resulted in a mix of younger working adults and older retired adults which likely contributed to the nonsignificant and negative association with bilateral temporal lobe thickness. A three‐ or four‐level ordinal variable may better represent work status. Finally, our investigation was limited to the socioenvironmental measures available. Numerous additional measures warrant consideration across different levels of analysis and influence including but not limited to experiences of discrimination, social support, infrastructure supporting health behaviors, and measures with consideration for the developmental stage of the experience (Alvidrez et al., 2019; Hill et al., 2015; Juster et al., 2011). Finally, analyses evaluating if socioenvironmental factors mediate the effect of chronic pain on cortical thickness would be particularly informative.

## 5 CONCLUSIONS

Determining the influence of socioenvironmental factors on health outcomes is essential to reducing health disparities and improving health for all. Socioenvironmental factors are associated with chronic pain and brain structure/function (Elbejjani et al., 2017; Mickle et al., 2023; Peters, 2006). Importantly, although socioenvironmental factors were not independently predictive in the adjusted analyses, with the inclusion of an array of socioenvironmental factors, previously observed sociodemographic (ethnic/race) groups, that is, NHB and NHW adults, bilateral temporal lobe cortical thickness differences were no longer observed (Tanner et al., 2021). Additionally, an additive inverse relationship was demonstrated between greater socioenvironmental risk and higher chronic pain stage and thinner temporal lobe brain structures. Our findings elucidate factors that may help explain observed disparities in chronic pain, ADRD risk, and bilateral temporal lobe brain structures.

## AUTHOR CONTRIBUTIONS

**Lisa H. Antoine**: Writing—original and final draft; formal analysis; conceptualization; methodology. **Jared J. Tanner**: Formal analysis; conceptualization; writing—original and final draft; methodology; supervision. **Angela M. Mickle**: Conceptualization; formal analysis; writing—original and final draft; methodology. **Cesar E. Gonzalez**: Data curation; writing—review and editing. **Daniel A. Kusko**: Data curation; writing—review and editing. **Kristen Allen Watts**: Data curation; writing—review and editing. **Deanna D. Rumble**: Formal analysis; writing—review and editing. **Taylor L. Buchanan**: Writing—review and editing. **Andrew M. Sims**: Formal analysis; writing‐review and editing. **Roland Staud**: Data curation; writing—review and editing. **Song Lai**: Data curation; writing—review and editing. **Hrishikesh Deshpande**: Data curation; writing—review and editing. **Brandis Phillips**: Formal analysis; writing—review and editing. **Thomas W. Buford**: Writing—review and editing. **Edwin N. Aroke**: Writing—review and editing. **David T. Redden**: Formal analysis; writing—review and editing. **Roger B. Fillingim**: Data curation; writing—review and editing; project administration. **Burel R. Goodin**: Conceptualization; data curation; formal analysis; writing—review and editing; methodology; supervision; project administration. **Kimberly T. Sibille**: Conceptualization; data curation; formal analysis; writing— original and finaldraft; methodology; supervision; project administration.

## FUNDING INFORMATION

NIH/NIA Grants R01AG054370, R01AG054370‐05S1, R37AG033906, and 5K02AG062498; UF CTSA Grant UL1TR001427, UAB CTSA Grant UL1TR001417, and NIH/NIGMS Grant 5K12GM088010‐12; NCATS Grant UL1 TR000064; McKnight Brain Institute: NSF DMR‐164479 and State of Florida

## CONFLICT OF INTEREST STATEMENT

The authors declare no conflicts of interest.

### PEER REVIEW

The peer review history for this article is available at https://publons.com/publon/10.1002/brb3.3330.

## Data Availability

Data will be made available by written request to the corresponding author.
